# Lectures on the Morbid Anatomy, Nature, and Treatment of Acute and Chronic Diseases, Delivered in the Theatre of Anatomy, Webb Street

**Published:** 1834-04-01

**Authors:** 


### MEDICAL QUARTERLY REVIEW.

### REVIEWS.

*Lectures on the Morbid Anatomy, Nature, and Treatment of Acute and Chronic Diseases; delivered in the Theatre of Anatomy, Webb-street, by the late* John Armstrong, m.d. &c. Edited by Joseph Rix, m.r.c.s.—London, 1834. 8vo. pp. 851.

Among the posts of honour which our profession offers to quicken the diligence of its most ardent votaries, there is no one which can vie in brilliancy with that of a popular lecturer. The applause which to others is doled out in scanty fragments, for whose complement they must appeal to the uncertain judgment of posterity, is meted out to him with an unsparing hand; and, even if he does not altogether escape unscathed by contemporaneous censure, he has but to enter his class-room "to read his glories in his pupils' eyes," and to hear the verdict of envy triumphantly cancelled by a boundless majority.

"Tunc dolor, et curæ, rugaque frontis abit."

In fact, the fame of which other men see but the reflected light through the long vistas of time and distance, is enjoyed by him face to face; so that for him the extravagant wish of the lovers in the old tragedy, that, for their sake, time and space might be annihilated, is almost literally fulfilled. This reward is great, but it is the reward of genius alone. He who hesitates in speech or in thought,—who reads his lectures, or compiles them,—who is too facile to have an opinion, or too obstinate to change one,—he who lacks the animation which brightens the features and enriches the voice of the discoverer and inventor, may be a trustworthy practitioner, but can never be a first-rate lecturer. The sparkling eye, the ever-varying tones, the pointed story addressed to the many, the sly allusion to the few, the original phraseology, the swelling period, the generous burst of enthusiasm,

—these constitute not the ornaments, but the very essence, of a course of lectures; without them, it becomes a mere book, with the unpleasing difference, that the price is measured by guineas instead of shillings.

The very pleasure with which we listen to the genuine lecturer arises in part from the instinctive consciousness that what we hear can never be read, since the most faithful report will be but a lifeless cast from the energies unfolded before us, and will indeed display but a very small part of them. No reasonable person, therefore, professes to be disappointed when he finds that a book can give, as the phrase goes, merely "the substance of lectures," and that the gorgeous colouring with which genius delights to invest its creations is gone for ever. In the present instance, however, the substance is so excellent, that we congratulate all our readers on the appearance of the volume before us: to the student it will be invaluable, and we know no one so advanced that he may not derive profit from the instructions of that master in the art, Dr. Armstrong. The chief merits of this system of physic consist in the minute plan for the examination of patients, the excellence of the diagnosis, and the simplicity of the treatment. This last indeed is carried so far, that to some it may seem to approach that undefined line which separates a merit from its kindred fault.

In the first lecture, when discussing the progress of medical science, Dr. Armstrong falls into mistakes which we should call very strange, if it were not that they are very common. After quoting Bacon, who says that up to his time but one man, Hippocrates, had studied physic the right way; and, after making the physician of Cos a standard by which to judge the merits even of Sydenham himself, we come to such errors as these:

"If we pass on from Egypt to Greece and Rome, where the cultivation of the fine arts was carried to the highest perfection, we shall find that but little progress was there made in the study of medicine; and it may be interesting to trace the causes which retarded the progress of this most important science.

"The first was ignorance: the ancients knew scarcely anything of anatomy and physiology. Their horror of dissection kept them in a state of profound ignorance of anatomy. Their physiology and pathology, for the same reason, were mere conjectures, and their conjectures were made without good foundation. Rather than wander in doubt, the human mind will always rest in error." (P. 2.)

So that Hippocrates, a few lines after being a standard of professional knowledge, becomes one of the makers of little progress, one of the profoundly ignorant of anatomy! The plain truth is, that those who accuse the ancients of ignorance of anatomy shew their own perfect ignorance of the books which they are libelling. Who is there who, when reading Celsus, was not struck with the passage in which he gives the principles of those "qui rationalem medicinam profitentur?" After telling us that they judged the study of physiology necessary to the practice of physic, he goes on to say: "Præter hæc, cum in interioribus partibus et dolores et morborum varia genera nascantur, neminem putant his adhibere posse remedia, qui ipsas ignoret. Necessarium ergo esse incidere corpora mortuorum, eorumque viscera atque intestina scrutari; longeque optime fecisse Herophilum et Erasistratum, qui nocentes homines, a regibus ex carcere acceptos, vivos inciderint, considerarintque etiamnum spiritu remanente, ea, quæ natura ante clausisset, eorumque positum, calorem, figuram, magnitudinem, ordinem, duritiem, mollitiem, lævorem, contactum; processus deinde singulorum et recessus, et sive quid inseritur alteri, sive quid partem alterius in se recipit. Neque enim cum dolor intus incidit, scire quid doleat, eum, qui, qua parte quodque viscus intestinumve sit, non cognoverit: neque curari id, quod ægrum est, posse ab eo, qui, quid sit, ignoret. Et cum per vulnus alicujus viscera patefacta sunt, eum, qui sanæ cujusque colorem partis ignoret, nescire quid integrum, quid corruptum sit; ita ne succurrere quidem posse corruptis. Aptiusque extrinsecus imponi remedia, compertis interiorum et sedibus et figuris, cognitaque eorum magnitudine: similesque omnia, quæ posita sunt, rationes habere. Neque esse crudele, sicut plerique proponunt, hominum nocentium, et horum quoque paucorum suppliciis, remedia populis innocentibus seculorum omnium quæri."—Lib. i. cap. i.

Now, without defending the excessive zeal of these ancient dissectors, or lauding them for cutting their way to knowledge through living flesh, we must say, that it seems most flagrantly unjust to accuse them of lukewarmness in the cultivation of anatomy: it is as if Venus had been accused of being a prude. We hope, however, that the increasing study of the old writers among the rising generation will prevent even the most ordinary minds from being seduced by fancies like these, though they have led astray a Lawrence and an Armstrong.

We have already observed, that the preliminary part in which our author teaches the method of investigating disease is extremely good, and the following quotations will, we think, support our opinion. They are taken from the 7th

Lecture, in which Dr. Armstrong gives the indications of a sound and of a morbid condition of the sanguiferous system.

"Celsus makes an important observation with regard to the pulse, and one which every medical man should recollect, especially in visiting females. If a medical man, for example, at his first visit to a female, feel the pulse, he will often find that it will be 100, 120, 130, or even 160, with no other bad symptom. In these cases you should invariably follow the rule laid down by Celsus.

"The moment a medical man enters the room to visit a female she pants and heaves at the chest, and the pulse becomes very quick. But after a time the respiration becomes tranquil, and the pulse becomes natural.

"Celsus says you should always feel the pulse twice, when you enter the room, and again before you leave it.

"If you judge from the first impression alone you will very often be deceived.

"And then certainly a long face has the effect of quickening the pulse; a solemn aspect often frightens women dreadfully, the heart pants and the pulse becomes quicker.

"The pulse may be quickened from organic disease affecting the heart, from tubercles in the lungs, or from extreme morbid sensibility of the nervous system.

"All persons who have extreme sensibility of the nervous system have a very rapid pulse. Women frequently complain of a pulse all over, at every part of the body. This depends upon the nervous system operating on the minute capillary vessels so as to produce an universal sensation of pulsation.

"Copious abstraction of blood will quicken the pulse even of a person in health; and if you bleed a person in health to-day, to-morrow, the next day, and go on thus, you will produce fever, and on the fourth day the blood will be covered with a thick buffy crust, or, as it is called, the inflammatory coat.

"Again, general debility quickens the circulation very much; and this may often be perceived in weak convalescents.

"A convalescent patient lies in bed, and desires, day after day, that he may be allowed to get up. At one visit the medical practitioner finds the pulse, in the recumbent posture, as slow as 60, and allows the individual to get up, and at the next visit he finds him perhaps sitting up by the fire, with a pulse of 120, or even as high as 160.

"When the pulse becomes thus quickened in the erect posture never allow a weak convalescent to sit up long; if you do, the heart partaking of the general weakness, and thus having the frequency of its action increased, the patient is sure to have a relapse of fever.

"The rule with regard to convalescents is this: if when a convalescent is sitting up, you find him with a slow pulse, he is safe; but if you find him with a pulse weak and quick, you must lay him flat, and never allow him to sit up more than a quarter of an hour or half an hour for the first, second, third, or fourth time, so as to accustom him to it gradually.

"The pulse may be preternaturally *slow*.

"When you have known the natural frequency of the pulse of an individual to have been in health 70, and being called to visit him find the pulse as low as 50 or 60, and especially if it be labouring and irregular, you may suspect that there is some mischief either in the brain, the lungs, or the heart, and should investigate the case accordingly.

"You should ascertain first, whether the patient has been taking any medicine which may account for the slowness or irregularity of the pulse. I have been called several times to patients in whom the pulse has been reduced very much by the daily exhibition of digitalis, and has become very small. The same thing may occur from the continued use of antimonials.

"Sometimes it is from the exhibition of opium, the opium having gorged the brain with blood, which has produced this change in the heart's action." (P. 80.)

"The first kind of irregular pulse may be called:

"1st. Oppressed.

"This is a kind of pulse which is very difficult to describe in words, although it is so peculiar I can readily recognise it. It gives one the idea as if the heart were struggling to throw off some superincumbent weight, or as if a weight were pressing upon a spring, which was reacting to endeavour to throw it off. This state of pulse frequently precedes apoplexy. It frequently depends upon congestion of the heart, the lungs, the brain, or the liver. It almost invariably happens that this oppression or obstruction is removed by bleeding, and it seems to indicate, as it were, the necessity for it.

"The pulse may also be,

"2d. Intermittent.

"When it is perfectly regular, you have sixty or seventy strokes in a minute. But when it intermits, you may count the pulsations, say, one, two, three, four, and then you lose the fifth; there is an intermission of the pulse between perhaps the fourth and the sixth beats. In other cases the intermission is at the tenth, twelfth, or twentieth beat, so that in fact there is a loss of a stroke ever and anon.

"This intermission of the pulse attends various conditions.

"I recollect that when I was a young physician this would have alarmed me exceedingly, for I thought it must depend upon organic disease. But now, if I were to observe an intermittent pulse, I should set about investigating the circumstances accompanying it.

"It is extremely common to meet with it in weak convalescents, on sitting up, from the heart partaking of the general debility; and in these cases it is of no consequence provided you do not allow the individual to sit up too long. In these instances you should be careful, as it were, to secure the strength by avoiding all demands upon it; by which means, and by a little wine, with a nutritious diet, this state of the pulse will be removed, together with the debility.

"Another frequent cause is disorder of the stomach, liver, or colon: indeed, this is the most common cause.

"When it occurs with a furred tongue, unnatural stools, or with overloaded large intestines, be careful not to pronounce an opinion that it depends upon organic disease; for the intermission of the pulse will cease by removing the disorder of the stomach, of the liver, or of the bowels. If the tongue be loaded you need hardly ever fear that this symptom depends upon organic disease.

"I know many persons, especially ladies, whose pulse becomes thus disturbed from a foul stomach, from an overloaded colon, or from a disordered state of the liver.

"Many medical practitioners have a pulse thus disturbed without any organic disease. A medical man, for example, finds that his pulse intermits, and he becomes very mnch alarmed at it; whereas, if he had observed it in any other individual under the same circumstances, he probably would not have been in the slightest degree frightened at it.

"But sometimes an intermittent pulse does attend organic affections of the heart, and then you will have other symptoms which indicate such organic change. If you come to observe minutely, you will find that in this case there are very distinct symptoms of organic affections, which are entirely absent when the intermission depends upon any other cause. The late Dr. Baillie thought that adhesions in the bag of the pericardium often were a cause of an intermission in the pulse. Though I entertain great respect for the opinions of Dr. Baillie, yet I believe that in this opinion he was mistaken: at least, I have seen several instances of snch adhesions in individuals in whose pulse during life I never had observed any intermission; and I believe that if it be a cause of an intermittent pulse, it is an extremely rare one." (P. 86.)

"Sometimes the sound of the heart is changed (the stroke of the heart being also changed,) in strength, in extent, and even in kind.

"If the substance of the heart be very much increased the extent of the sound is much greater than natural; it is much stronger than natural, and it emits a different sound. If the substance of the heart be thinner and softer than natural, the sound is more clear, more soft, and more fluent, than natural.

"If the valves of the heart be ossified there is a very peculiar sound, which Laennec compares to the sound produced by the compression of a pair of bellows. I have often heard this sound produced by the whizzing of a toy which boys have, and which is called a whiz-gig. Sometimes there is a cooing noise, like that of a turtle dove, very distinct.

"A gentleman occasionally calls on me whose heart emits a sound very much like that of the cooing of a dove. I have no doubt that he labours under enlargement of the heart with ossification of the valves. The sound is so distinct, that if I lay my hand over the region of the heart I can hear this cooing noise, which is conveyed along my arm to my ear. It is just like what you would imagine the cooing of the turtle dove would be if confined within the chest. Now, if this were a poor man, who had a mind to make his fortune, he might easily make all the old women believe that he really had a live turtle dove within him.

"In certain organic affections of the heart sometimes there is a very peculiar feel, an emphysematous feel, in the carotid and subclavian arteries; it is also felt in the wrist. The artery feels exactly as if air were collected in the cellular connecting membrane of its coats. Laennec supposes that air really is so collected. When it occurs, it generally attends organic affection of the heart, but not always." (P. 89.)

In the next Lecture, on the sound and morbid condition of the "Concoctive and Absorbent System," Dr. Armstrong has many valuable observations on the appearance of the stools; some of them will be particularly useful to practitioners afflicted with the mercurial monomania, who, Hahnemann-like, give calomel to cure what calomel has caused; but, un-Hahnemann-like, give it in doses which are by no means infinitesimal.

"You must take into account the natural colour of the stools. You should always consider the healthy state, and contrast it with the state that exists, in order to know whether there be any morbid change.

"Therefore I repeat, you must first ascertain the natural colour of the evacuations. Some have described it as the colour of rhubarb wetted with water, others have compared it to turmeric, and some to virgin gold; but the best way is to look at it if you wish to know its natural colour.

"You must remember that if an evacuation be kept for some time it becomes very dark indeed from exposure to the air in an open vessel; and, unless he took this circumstance into account, a medical man might be very apt to be deceived as to the healthy condition of the evacuations.

"The consistence of the evacuations is of importance in many cases.

"There is a certain healthy consistence, and any deviation from that consistence is a sure indication of something wrong. There is a natural smell in a healthy evacuation which is peculiar.

"All these, then, are very important: and even,

"2d. The size of the evacuations is very important.

"Sometimes you may detect stricture or piles from an unnatural size of the evacuations. In a permanent stricture of the rectum the evacuations are almost invariably twisted like a corkscrew; but recollect that the same also occurs occasionally from a temporary stricture.

"3d. The colour of the stools is important.

"When there is a deficiency of bile, the evacuations sometimes are of the colour of lime, of pipe-clay, or of slate.

"When there is an excess of bile, the stools are deeply tinged of a yellow colour, and are generally looser than natural.

"The bile may be depraved, and then the colour varies; sometimes it is as dark as tar; and then it may be distinguished from blood by holding it to the light, or by mixing it with water.

"Or the evacuations may be like melted resin. This very often is the case in typhus fever.

"Or sometimes they are green in colour from the same cause.

"You must take into account the influence of diet, and the influence of certain medicines, upon the colour of the evacuations.

"If an adult live upon a milk diet there will be an apparent deficiency of bile in the evacuations; and if you were not aware of this circumstance you might presume that the liver was disordered, and put the individual very unnecessarily under a course of mercury. So too, if you inferred from the green appearance of the evacuations that the liver was disordered so as to require the use of mercury, you might be mistaken, for this green colour of the stools might be produced by mercury. A glairy green appearance of the stools is very frequently indeed produced by mercury.

"Again, a curdled appearance of the stools is a very common effect of the exhibition of mercury. Another kind of stool which is very common from mercury is one which very much resembles mock-turtle soup.

"Now you might think that this is of no consequence, but it really is of immense importance, especially in London. For instance, a dose of mercury is given, and the stools are examined, and to some men the conclusion is irresistible, that because the stools are of a morbid appearance mercury must be requisite to correct them. A medical man, therefore, exhibits mercury for a supposed disorder of the chylopoietic viscera, especially the liver; and thus the unnatural state of the stools, which was first produced by mercury, is afterwards maintained by mercury. I recollect I once saw a lady who had been for several months undergoing courses of mercury; the blue pill was continued to correct the stools, till she was reduced to a skeleton, and was rendered so nervous that an angry look made her shed tears, and if the door were suddenly opened she started, and was excessively agitated. The blue pill was left off, and the natural appearance of the stools soon returned. I recollect I saw a case in which four grains of calomel were given every six hours, and produced stools very much resembling mock-turtle soup; I told the medical man that the exhibition of the mercury was the cause of the unnatural stools. The mercury was left off, and the stools again became natural.

"If you neglect to pay attention to this circumstance you may entirely destroy the health of an individual. Thus the exhibition of mercury day after day, with a cool skin, is one of the most destructive practices with which I am acquainted. There is now a work, written *ad captandum*, in which it is laid down as a rule, that mercury is to be continued as long as the stools are unnatural. If you were to follow this advice, and give mercury day after day, and week after week, you would very often commit a serious error, and ruin the health of your patient.

"If you have any doubt of the cause of the unnatural appearance of the evacuations in any case, omit the mercury, and omit all medicines for a few days, and see whether it depends upon the exhibition of mercury.

"If any man in health have his mind constantly and entirely engaged, you will find that the stools will undergo a great change. No man can have observed the influence of mental emotions upon the functions of the body, without having noticed the changes which the state of the mind produces in the secretions and excretions—as the fæces.

"The incautious and indiscriminate use of mercury is a great and a growing evil, in this town especially, where there is as much fashion in physic as in fitting-out the shops in Bond-street.

"There are also other medicines which produce a change in the appearance of the stools.

"Iron changes the colour of the evacuations to black.

"A friend of mine cured an hypochondriac patient by turning his stools black with iron. This patient had been from one doctor to another, but still he said he was no better. My friend said to him, 'These doctors have all entirely mistaken your case. I can very soon cure you. Your complaint arises from some black matter in your bowels, which wants to be purged away.' He gave the patient some sulphate of iron, and made his stools as black as ink; and then he gave him a purgative, and told him to look at his stools, and see whether the black matter had come away. The patient was so delighted with his black stools, that, as soon as he saw them, he ran to the medical man, and said, 'Doctor, I have passed it—I have passed it—as black as my hat! and now I am cured!'

"Sulphur changes the stools very remarkably; it darkens them.

"Senna darkens the evacuations.

"Spinach renders the evacuations green; and, if you did not happen to know the influence of different kinds of food upon the stools, you might form an erroneous opinion from the examination of the stools after an individual has been eating spinach.

"Some wines influence the colour of the stools. Claret gives a very peculiar laky tinge to the fæces. If you wish to know precisely the colour which it produces in the stools, take a few glasses or a bottle. You will find claret a very nice wine." (P. 100.)

Our author's account of congestive fever is exceedingly good; and the interest of the following extracts must be doubled by the reflection, that the Asiatic cholera itself is but a violent form of this disease.

"Whenever you are called to a patient in the extreme shock of an accident, with a pale skin, with a sunk countenance, with a feeble pulse, and with a weak respiration, do not bleed him at all, but give him a little wine or brandy. A friend of mine was called to a young lady in this state, about three weeks ago; the friends of the lady urged him to bleed her, but he refused, and told them that if he bled her she would die. On the contrary, he gave her diffusible stimuli; and he did perfectly right. It is by far too common for medical men to abstract blood immediately they are called to an accident; but the first extreme shock should be past before bloodletting is resorted to. One gentleman, whom I saw frequently in the country, used to treat common congestive fever, first by brandy, secondly by the hot-water bath, and thirdly by bleeding. He was a very good practitioner, but his ideas passed so rapidly through his mind, that he had not time to analyse them. His practice was to put the patient into a hot bath with salt, and during the time he was in the bath to give him brandy, and then bled him. Now this is not bad practice. He in fact created a degree of excitement before he abstracted blood; and then he bled with great caution: when he had once created excitement, then he ceased to use stimulants, either internal or external; and that is the proper plan to adopt.

"I saw a gentleman one morning who had an attack of the extreme form of common congestive fever. His surface was universally pale and cold; he had an intoxicated expression of countenance; when lifted, he dragged his limbs after him as if they were paralytic; his lip and cheek, together with the state of the respiration, showed an extreme congestion in the lungs and bronchial lining; he had also copious purging and vomiting; in short, he had congestion in the brain, in the bronchial lining, in the lungs, and in the liver; and laboured under what would be called an attack of cholera morbus. The attack came on at seven o'clock, and I saw him at eight; and I am confident he would have died in an hour or two more. All the ordinary means had failed to create excitement; brandy, opium, and so on, had been tried; and then I sent for a hot-air bath. In half an hour after its application the surface became universally warm, and he was perfectly convalescent.

"I attended a young lady who was attacked with giddiness, universal and oppressive debility, vomiting, and diarrhœa. When I saw her she looked like a person intoxicated; the tunica conjunctiva was blanched, the face pallid, the surface of the body cold, the respiration weak and impeded, and the lips were blue; she had no muscular power; the head rested on her shoulder, and the hands were by her sides. I placed bottles of hot water to the feet, a bladder of hot water to the stomach, and gave her hot water and opium internally. Nothing, however, was of benefit, and it was apparent that she was rapidly sinking. In this case I sent for a hot-air bath, which was immediately applied; and in half an hour the pulse rose and was bounding, her countenance became animated, and she was nearly convalescent. Nothing further was required but the exhibition of slight calomel purges. This, according to our nosologists, would be called cholera morbus, but it was a case of congestive fever; and in these cases, if assistance be not promptly rendered, death will be the consequence: the blood will coagulate in the interior of the body.

"I saw another individual, who was brought into the Fever Hospital, who was sinking very rapidly from an extreme form of congestion of the lungs, and of the bronchial lining; and the hot-air bath produced a state of excitement almost immediately, and by following this up by small doses of calomel and opium, he did perfectly well.

"Just before lecture one evening I was called to a young lady, who had been out all the morning, and was very cold, and as soon as she returned home ate a piece of cold apple dumpling. She had an idiotic expression of countenance; she was stupid, and had a flow of saliva from the mouth; the respiration and heart's action were impeded, and her skin was universally cold. I directed the exhibition of an emetic, which did not operate; but by the application of the hot-air bath all these symptoms were removed with very great rapidity, and on my return I found her convalescent.

"I recollect I saw a young man who laboured under an extreme form of congestion in the spinal cord; he was struggling in convulsions, with a clear head, and with a feeble pulse; and the symptoms in this case were rapidly removed by the use of the hot-air bath. This individual was afterwards preserved several times from an attack of congestion by the administration of drachm-doses of the tincture of opium. * * *

"When offending ingesta produce congestion life sometimes depends on the exhibition of an emetic. You read in the papers of cases of apoplexy arising from this source; but the fact is, the stomach being disturbed, the heart's action sinks, and, by consequence, the flow of blood from the brain is retarded, and the patient falls down pale and insensible, and sometimes dies as if he were shot. In some cases the person may live in this state for hours.

"In extreme cases the best emetic is sulphate of zinc. I saw a case in which it operated almost like a charm.

"A gentleman who was travelling ate some cold veal pie, and fell on the floor in a state of extreme congestion, with the heart extremely oppressed. A large dose of sulphate of zinc was given, and all the symptoms were got rid of by vomiting.

"In these cases sometimes emetics do not operate; and, when the skin is universally cold, the pulse feeble, the respiration oppressed, and the prostration of strength very great, life may be saved by half a glass or a glass of dry brandy, with twenty, thirty, or forty drops of tincture of opium. Sometimes the pulse is struggling, the respiration anxious, the intellect clear, and the heat of the skin nearly natural; and then brandy is useful. If no emetic be at hand, introduce your finger into the throat, or tickle the fauces with a feather. A teaspoonful of mustard given in tepid water will cause vomiting, or oil may be given for that purpose. These cases are often succeeded by inflammation, and therefore you should attend closely to the patient for a day or two. When a large quantity of spirit has been taken, the patient sometimes falls in the same way. If the pupil contract on the application of light, and an emetic operate, the patient generally recovers. If emetics fail, apply the instrument. An elastic bottle has long been used for this purpose." (P. 164.)

The preceding observations apply to the extreme form of congestive fever: the intermediate form bears bleeding in the commencement; but still "never bleed a patient to syncope in congestive fever; for, if you do, he may die under that state. It is better to stop while you have a pretty round pulse. And here I may remark, that in cases of organic affection of the heart you should never bleed to syncope." (P. 170.)

When speaking of the mild form of the same disease, our author says,

"One point of very great importance is the chill of the surface. If you investigate the history of examples of acute and of chronic diseases of a serious character, you will find that many of them were preceded by an universal chill of the surface, which chill will very often be found to have continued a great many hours. This takes place in many of the cases of congestive, of simple, and of inflammatory fever, which occur in this country, from the low and variable temperature of our atmosphere. And no rule is more important to the public than that which instructs them to restore the natural heat of the surface as early as possible. I generally mention this to all the families whom I attend. I advise them, if ever they be chilled, to make for the first hot bath they know of, and restore the heat of the surface.

"The chill very often continues for many hours before dangerous venous congestion comes on; or before excitement, the consequence of which often is an attack of inflammatory fever, occurs; and, by the use of a hot bath, you may often prevent a great deal of mischief. The temperature of the bath in these cases seldom ought to be less than 100° Fahr. In Paris you may have a hot bath in any house at five minutes' notice. In every well-regulated government there ought to be a Minister of Health. There are many things in London the effects of which are very injurious to the public welfare; and the notorious deficiency of warm baths is one of them.

Besides this there are a great many others which I could mention." (P. 172.)

It must be confessed, that the deficiency of baths, and the neglect of bathing, in this country, are most reprehensible; and, as in many other instances, society seems confined in the iron limits of a vicious circle: there would be more baths, says A., if there were more bathers; no, replies B., if there were more baths, there would be more bathers. Any one who could afford the risk, and would set on foot half-a-dozen establishments where a warm bath could be procured for a trifle, would be a benefactor to his country. A great evil also arises from bathing being considered synonymous with cold bathing: thousands who dislike the cold bath, and have been told that the warm bath is "relaxing," go about, in consequence, in contravention of the laws of Hygiene, with the skin in an unwashed, untranspiring state.

In speaking of the morbid anatomy of the pleura, Dr. Armstrong says very correctly, "Partial adhesions often take place from effusions without pain, the consequence of slight inflammation, which is not perceived. You will scarcely examine any person above forty years of age, in whom there is not some adhesion of the pleura, although perhaps through life there was no complaint of symptoms of inflammation of the chest." (P. 220.) Does not this well-known fact shew almost to demonstration the inutility of bleeding in very slight inflammations, which get well of themselves? In these pleuritic cases, which occur by myriads, the disease neither injures the constitution, nor permanently affects the respiration; yet, if detected during the life of the patient, would almost inevitably entail upon him some very active treatment.

Our author enters at length into the relative advantages to be derived from the different modes of abstracting blood. He dislikes cupping, which he calls barbarous, and supposes (erroneously, we think,) to be equivalent in its effects to venesection: the application of leeches he extols.

"There seems to be something peculiar in the effects of leeches, especially when the heat on the surface is not very high, and the heart's action not very much increased. My attention was drawn to this peculiarity of effect accidentally.

"I ordered a gentleman to be bled for a pulsating pain in the head, corresponding in frequency to the pulsations of the radial artery. He was bled repeatedly for it without relief. I then ordered twelve leeches to the temple, and accidentally putting my finger on the radial artery, I found that the pulse had fallen twenty beats; though it had not fallen before under repeated large bleedings from the arm. The leeches completely relieved the pain in his head. I could not account for this, but I have observed it repeatedly since." * * *

"Leeches seem to have a far more powerful effect in relieving inflammation of the mucous membranes than either cupping or the lancet. They seem to have some specific influence on the heart's action, and, through it, on the circulation. This was remarkably displayed in my own case when I had an attack of dysentery; I was passing slimy and bloody evacuations every half hour; but, after the first three applications of leeches, I passed no blood at all in the stools. We have many similar examples of certain effects produced by the operation of certain medicines or remedies on certain parts of the body; thus, when the female breast is distended after delivery, the milk will disappear in a great measure if you give a purgative." (P. 324.)

Dr. Armstrong gives two cases in which the hemorrhage from leech bites was fatal; and he recommends that, when leeches are applied to infants, they should be applied over a bone, in order to facilitate stopping the bleeding by pressure. After mentioning several astringent applications, he says, "A very good styptic is made of equal parts of sulphate of iron and superacetate of lead." (P. 325.) These two astringents will decompose each other, forming acetate of iron and sulphate of lead.

It is painful to us to see our author, in various parts of these lectures, endeavouring to tear the laurel wreath from the brows of Cullen and Heberden; vainly supposing that his censures could cloud reputations which know no other limits than those of the civilised world! He accuses Cullen of having given a very faulty definition of typhus fever; and says, "indeed, we have nothing but words under the miserable system of Cullen." (P. 563.) The truth is, however, that Armstrong's and Cullen's definitions of typhus nearly coincide, excepting that our author supposes the disease not to be contagious. Dr. Armstrong moreover imagines typhus and ague to be kindred diseases, and to arise from the same cause. He also believes plague, yellow fever, and typhus fever, to be modifications of the same affection. The following is his account of the post-mortem appearances in typhus fever.

"If you cautiously examine bodies after fatal cases of continued typhus fever, you will find the following appearances:

"You will find, on cutting the brain, that it exhibits more bloody points than natural; that the pia mater is gorged with red blood; that the arachnoid membrane is milky or opaque in some places, and thickened; that there is some effusion of fluid, generally serum with loose coagulable lymph, between the membranes; and that the membranes of the spinal cord are in a similar condition; at least, as far as my examinations have gone, it has been the case. Of the state of the brain and its membranes I can speak confidently, having invariably found them affected in more than one hundred cases, without a single exception.

"The bronchial lining is invariably found highly congested with dark blood, and a sticky secretion which besmears the membrane exercises a most important influence over the pathology of the affection, by changing the constitution of the blood in a way which I have already explained. If the sticky varnish be washed off with a sponge, the membrane exposed to the air soon becomes vividly red.

"The liver generally contains more blood than natural, and a venous or arterial tree is found in the mesentery, when no calomel purges have been given. If calomel purges have been given, then the appearance of the liver is pretty natural.

"Some traces of inflammation are found invariably in the mucous membrane of the small intestines, and especially of the lower portion of the ilium. This portion of the ilium is invariably found inflamed, either with or without ulceration. When the affection has gone on for a fortnight or three weeks, you will almost invariably have inflammation and ulceration there, and the mesenteric glands will be more or less enlarged. When diarrhœa has existed the upper part of the colon also will be found inflamed.

"Occasionally the mucous membrane of the stomach is red, thickened, and pulpy.

"Sometimes it happens that the internal tunics of the arteries and veins are inflamed. This seems to be an accidental concomitant, and not a necessary or essential part of the affection.

"Sometimes, though rarely, the serous membranes are inflamed; but this too seems rather an accidental than an essential part of the pathology, if we except the arachnoid membrane of the brain, which I suppose we must consider as a serous membrane.

"The skin undergoes great changes: generally it is universally dry, and furfuracious, and more contracted than natural. When the internal mucous membranes are much affected, the functions of the skin are generally considerably disturbed also.

"Whatever be the age, whatever be the sex, and whatever be the constitutional peculiarities of the individual, there is this remarkable circumstance, that this malaria, when it brings about continued typhus fever, produces in all persons uniformly the same affection of certain parts, the same affection of the brain and its membranes; the same affection of the bronchial lining; the same affection of the mucous membrane of the small intestines; and the same affection sometimes of the mucous membrane of the stomach." (P. 564.)

In discussing the regiminal treatment of this disease, he tells us that

"Rush, who has been called the American Sydenham, mentions a very remarkable and interesting case, showing the influence over typhus fever which is produced by cheerful impressions on the mind. When a youth he was educated in the country, in a very remote part of which he was in the habit of visiting, in company with a farmer's daughter, various scenes of beauty and sublimity, and, among others, the nest of an eagle in a romantic situation. For some time these visits were very frequent. Rush afterwards left the school, and settled in Philadelphia, where he found his former associate a married woman. Many years after she had an attack of typhus fever, under which she lay in a complete state of insensibility, apparently lost to all surrounding objects. In this state Rush, then a physician, was called to visit her. He took her by the hand, and said with a strong and cheerful voice, 'The eagle's nest!' The words revived an association of ideas comprehending the actions of her youth. She immediately grasped his hand, opened her eyes, and from that hour recovered rapidly." (P. 573.)

Our author says,

"I have known several individuals excessively dissipated among women, who have, by adopting certain precautions, avoided both chancre and gonorrhœa. They were extremely cleanly, and after indiscriminate intercourse they used soap and water twice or three times, and two or three clean towels. I know one individual who had been several times the subject of gonorrhœa and chancre, but who never had an attack after he adopted this plan, although he continued equally dissipated. I think it is almost a complete preventive." (P. 763.)

We have sometimes thought that this precaution must be of more efficacy in preventing chancre than gonorrhœa; or, in other words, that the prevention of the latter disease must require very industrious and vigorous washing indeed, if it arises from particles of virus lodged in the urethra. Still it is an admirable preventive. Diluted liquor potassæ, which is often recommended, has one advantage: it is medicinal, and therefore more likely to be adopted.

It is almost needless to give a formal recommendation of a work like this. Our numerous quotations carry their own panegyric with them. Mr. Rix has our hearty thanks for the zeal and ability which he has displayed in enriching medical literature with this excellent text-book.

